# Preconditioning lessens high fat induced metabolic syndrome along with markers of increased metabolic capacity in muscle and adipose tissue

**DOI:** 10.1042/BSR20181873

**Published:** 2018-12-14

**Authors:** Songpei Li, Xiu Zhou, Eunjung Jo, Ali Mahzari, Sherouk Fouda, Dongli Li, Kun Zhang, Ji-Ming Ye

**Affiliations:** 1 School of Health and Biomedical Sciences, RMIT University, Melbourne, VIC, Australia; 2 School of Biotechnology and Health Sciences, Wuyi University, Jiangmen, China

**Keywords:** FGF21, high fat diet, metabolic disorders, obesity, preconditioning

## Abstract

Postnatal overconsumption of fat is believed to increase the susceptibility to metabolic disease in the later life. Here we examined whether prior exposure to high fat (HF) in the adulthood may also accelerate the development of metabolic disorders in mice. Adult mice (12 weeks) were pre-exposed to two episodes of an HF diet each for 2 weeks followed by 2 weeks of washout with a low-fat diet. The mice were then fed the same HF diet for 6 weeks. Unexpectedly, prior exposures to HF diet significantly alleviated body weight gain, visceral adiposity and glucose/insulin intolerance during the period of last HF feeding. These protective effects were evident without changing calorie intake and were specific for HF, but not high fructose (HFru) diet. Following the HF prior exposures was increases in plasma fibroblast growth factor 21 (FGF21), the expressions of phospho-AMP-activated protein kinase (pAMPK), mitochondrial complex II and the expression of uncoupling protein (UCP) 3 in muscle and UCP1 and Sirtuin 1 (SIRT1) in adipose tissue. However, in the liver there was no significant change in pAMPK, SIRT1 expression or the capacity of glucose production. These findings indicated that, instead of exacerbating metabolic conditions, prior exposures to HF diet lead to the preconditioning against subsequent overload of HF, possibly involving FGF21-associated enhancement of markers for metabolic capacity in muscle and adipose tissue. This paradoxical phenomenon may offer a unique paradigm to identify factors and explore dietary ingredients with beneficial effects for the control of the metabolic syndrome in humans.

## Introduction

Central obesity and insulin resistance are fundamental to the pathogenesis of the metabolic syndrome [1,2]. Increased consumption of high-calorie diets and decreased energy expenditure (such as in a sedentary lifestyle) are major risk factors of obesity [1,2]. One of the most important dietary compositions leading to obesity and insulin resistance is the high fat (HF) content in diet, particularly the lard-based HF diet [3,4]. However, it remains unclear that whether prior exposure to HF diets in adulthood has long-term metabolic effects after the cessation of the HF feeding.

There is emerging evidence to suggest that various environmental factors may have long-term impacts on the metabolic syndrome. A well-known example is the long-term benefits of an intensive glycaemic control for diabetic patients in a 10-year post-trial follow-up of the United Kingdom Prospective Diabetes Study [5,6]. In addition, caloric restriction for a period of time in adulthood has been shown to delay the aging and extend the lifespan in various animal models [7,8]. In humans, it has been reported that calorie restriction can induce long-term weight loss [9]. Based on these reports, it appears to be reasonable to speculate that HF feeding diet may have certain long-lasting effects on whole-body metabolism beyond the feeding period.

Indeed, overfeeding of postnatal rats can increase the risk of developing obesity and fatty liver disease in adulthood [10–12]. In analogue to this, obese children are more likely to develop obesity and associated metabolic disorders in their adulthood [13]. Therefore, we hypothesise that prior feeding with an HF diet in adulthood may exacerbate the metabolic syndrome when challenged with subsequent feeding of the HF diet. To test this hypothesis, the present study fed adult mice two episodes of HF diet and then re-fed the animals the same HF diet after the initial metabolic disorders was washed out with a chow (CH) diet. The metabolic response to the subsequent HF feeding was assessed by examining the adiposity and pre-diabetic indicators compared with those of the HF-fed mice without any prior exposure.

## Materials and methods

### Animal study

**Figure 1:**
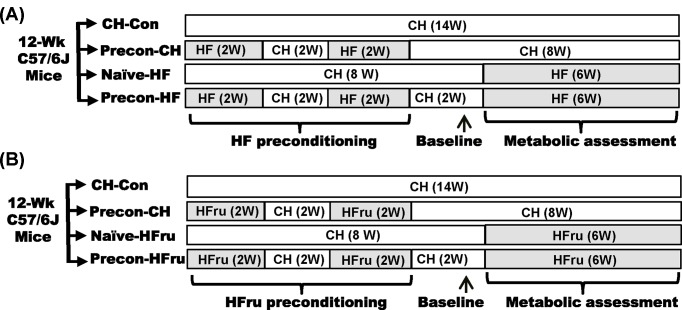
Schematic illustration of study design (**A**) Male C57BL/6J mice at age of 12 weeks were assigned to different dietary regiments to examine the legacy effects of preconditioning on subsequent exposure to the HF diet. Preconditioning was introduced twice with HF diet, each followed a washout period by a CH diet. Abbreviations: CH-Con, normal CH control mice (without preconditioning); naïve-HF, HF-fed mice without preconditioning; Precon-CH, CH-fed mice after HF preconditioning; Precon-HF, HF-fed mice after preconditioning with the same HF diet. (**B**) In a separate experiment, mice were fed either a HFru or CH diet with the same feeding episodes of the HF-preconditioning experiment to compare the effects of HFru prior exposures on subsequent exposure to the same HFru. Abbreviations: Naïve-HFru, HFru-fed mice without preconditioning; Precon-CH, CH-fed mice after HFru preconditioning, Precon-HFru: HFru-fed mice after preconditioning with the same HFru diet.

Male C57BL/6J mice (10 weeks old) were purchased from Animal Resources Centre (Perth, Australia) and housed at 22 ± 1°C on a 12-h light/dark cycle with free access to water and food. After acclimatisation for 2 weeks, mice were assigned *ad libitum* to four dietary regimens to examine the effects of HF prior exposures on subsequent exposure to the HF diet as illustrated in Figure 1A. The macronutrients of CH diet were composed of (in calories) 70% carbohydrates, 10% fat, 20% protein (Specialty Feeds, Australia). The HF diet consisted of 45% calories from fat (lard), 20% calories from protein and 35% calories from carbohydrate supplemented with 0.2% cholesterol [14]. In a separate experiment, mice were preconditioned with a high fructose (HFru) diet in the identical protocol to compare the effects of HFru prior exposures on subsequent exposure to the same HFru (Figure 1B). The calories of the HFru diet were made of 35% fructose, 35% starch, 10% fat and 20% protein [15,16]. Body weight (BW) and food intake were monitored three times a week. All experiments were approved by the Animal Ethics Committee of the Royal Melbourne Institute of Technology University (Project #1414) in accordance with the guidelines of the National Health and Medical Research Council of Australia.

### Assessment of metabolic response

The metabolic response of the preconditioning was assessed during the final six weeks of HF feeding. For glucose tolerance test (GTT), animals were fasted for 5 h and glucose (2.0 g/kg BW) was administrated to animals by intraperitoneal (ip) injection. Blood glucose levels were measured at 0, 15, 30, 60 and 90 min using a glucometer (Accu-Chek, Roche) whereas blood insulin levels were sampled at 0, 15, 60 and 90 min from the tail vein into heparin-coated capillary tubes (SteriHealth, #I-10086). The whole-body insulin sensitivity of mice was measured at week 11 by an insulin tolerance test (ITT) [17]. After 5 h of fasting, insulin (100 IU/ml, Novo Nordisk Novorapid Penfill) was diluted to 0.2 IU/ml with saline and injected intraperitoneally into the mice to reach a final dose of 0.75 IU/kg BW. Subsequently, blood glucose levels were measured at 0, 20, 40, 60 and 80 min using a glucometer. The capacity of hepatic glucose production was measured by ip pyruvate tolerance tests (ipPTT) [17,18]. Pyruvate (Sigma–Aldrich, #2256) was prepared in 1× PBS buffer. Pyruvate (2.0 g/kg BW) was administered to animals by ip injection after overnight fasting. Blood glucose levels were measured at 0, 15, 30, 60 and 90 min using a glucometer. At the end of the study, mice were killed by cervical dislocation after removing the food for 5–7 h. Plasma samples were collected from the tail tip before the killing of mice and stored at −80°C. Epididymal fat mass was weighed using an analytical balance. Liver and quadriceps muscle were free-clamped and stored at −80°C for subsequent analysis.

Plasma fibroblast growth factor 21 (FGF21) levels were measured using an ELISA kit from Hong Kong University Antibody and Immunoassay Services (#32180) following the manufacturer’s instructions. The effects on hepatic steatosis were assessed by measuring the triglyceride (TG) content in the liver. Liver TGs were extracted from the freeze-clamped liver tissue by the method of Bligh and Dyer [19]. Briefly, tissue samples were homogenised in a mixture of chloroform and methanol (2:1) using a glass homogeniser and placed on a shaker overnight. NaCl (0.6%) was added to extract the aqueous phase. The mixture was centrifuged at 1000×***g*** for 10 min and the lower organic phase was transferred to a new tube. The extract was then dried and redissolved in ethanol prior to the measurement of TG concentrations using a TG GPO-PAP reagent (Roche Diagnostics, #11730711216, Australia) [20].

### Measurement of mtDNA content

Total DNA, including mitochondrial DNA (mtDNA) and genomic DNA, was isolated from tissues using a DNA isolation kit from Invitrogen (#K1820-02). The DNA extract was quantitated and diluted to 2 ng/μl as the template for PCRs. Monocarboxylate transporter 1 (MCT-1) and NADH-ubiquinone oxidoreductase chain 5 (ND-5) were used as a marker of genomic DNA and mtDNA, respectively. The sequences of primers were: MCT-1 forward 5′-TAGCTGGATCCCTGATGCGA-3′; MCT-1 reverse 5′-GCATCAGACTTCCCAGCTTCC-3′; ND-5 forward 5′-GCAGCCACAGGAAAATCCG-3′; ND-5 reverse 5′-GTAGGGCAGAGACGGGAGTTG-3′.

### Measurement of citrate synthase and β-hydroxyacyl-CoA dehydrogenase activity

Citrate synthase (CiS) and β-hydroxyacyl-CoA dehydrogenase (β-HAD) activities were measured as previously described [16]. Frozen samples were homogenised in a buffer (pH 7.4) containing 175 mmol/l KCl and 2 mmol/l EDTA. For CiS activity, tissue homogenates were mixed with a working solution containing 450 μM acetyl-CoA (Sigma–Aldrich, #A2056) and 0.1 mM 5,5′-dithiobis(2-nitrobenzoic acid) (DTNB, Sigma–Aldrich, #D218200), and absorbance was measured at 405 nm as blank values. Oxaloacetic acid (Sigma–Aldrich, #O4126, final concentration 1 mM) was then added to the assay system to initiate the reaction. The enzyme activity was calculated based on the slope of the absorbance curve. For β-HAD activity, tissue homogenates were mixed with a working solution containing 0.2 mM NADH (Sigma–Aldrich, #N4505) and absorbance at 340 nm was measured as the blank. Acetoacetyl-CoA (Sigma–Aldrich, #A1625, final concentration 0.1 mM) was then added to the assay system to initiate the reaction. The enzyme activity was calculated based on the slope of the absorbance curve and the extinction coefficient of NADH (6.22 µmol^−1^.cm^−1^).

### Western blotting

Western blotting was performed as described previously [21]. Proteins prepared in Laemmli buffer were separated by SDS/PAGE, then transferred to PVDF membranes (Bio-Rad, U.S.A.) and blocked in 3% BSA. Membranes were probed with the following primary antibodies. Anti- total- and phospho- AMP-activated protein kinase (AMPK), anti-sirtuin 1 (SIRT1), anti- total- and phospho- acetyl-CoA carboxylase (ACC), anti-glyceraldehyde 3-phosphate dehydrogenase (GADPH) and anti-tubulin antibodies were purchased from Cell Signaling (U.S.A.). Anti-mitochondrial respiratory complexes and anti-PGC1α antibodies were obtained from Abcam (U.K.) and Merck Millipore (AU), respectively. Anti-uncoupling protein (UCP) 1 and anti-UCP3 antibodies were purchased from Santa Cruz Biotechnology (U.S.A.) and ThermoFisher Scientific (U.S.A.), respectively. Western blot membranes were incubated with corresponding secondary antibodies from Santa Cruz Biotechnology (U.S.A.) that were conjugated to horseradish peroxidase (HRP) and developed using ECL HRP substrate from PerkinElmer (U.S.A.). Images of the membranes were taken with the ChemiDoc system and densitometry analysis was performed using Image Lab software 5.0 (Bio-Rad Laboratories, U.S.A.).

### Quantitative real-time PCR

RNA was extracted using TRIzol reagent (Invitrogen, #15596026) and genomic DNA was digested using amplification grade DNase (Invitrogen, #18068-015, Australia). RNA extract was reverse-transcribed using a High Capacity cDNA Reverse Transcription kit (Applied Biosystems, #4368814, Australia) according to the manufacturer’s instructions. Primers (GeneWorks, Australia) and SYBR green supermix (Bio-Rad, #170-8880, U.S.A.) were used for quantitative real-time PCR using QIAGEN Rotor-Gene Q PCR system (Germany). *18s* was used as the normalising control gene and results were analysed by the ΔΔ*C*_t_ method. Sequences of the primers were: PGC1α AAACTTGCTAGCGGTCCTCA (forward) and TGGCTGGTGCCAGTAAGAG (reverse); UCP3 TGTCAACCAACTTCTCTAGGATAAGG (forward) and CACTGTTGTCTCTGCTGCTTCTG (reverse); and 18S CGCCGCTAGAGGTGAAATTCT (forward) and CGAACCTCCGACTTTCGTTCT (reverse).

### Statistical analyses

Data were expressed as means ± S.E.M. The individual results were screened for their distribution patterns prior to the statistical tests. A Student’s *t*test was performed for the comparison of normal distributed data. Data of FGF21 did not follow a normal distribution and thus statistical comparisons were made using a nonparametric test for independent samples (SPSS statistics 23). Differences at *P*<0.05 were considered statistically significant.

## Results

### Effects of HF prior exposures on HF-induced BW gain and adiposity

**Figure 2:**
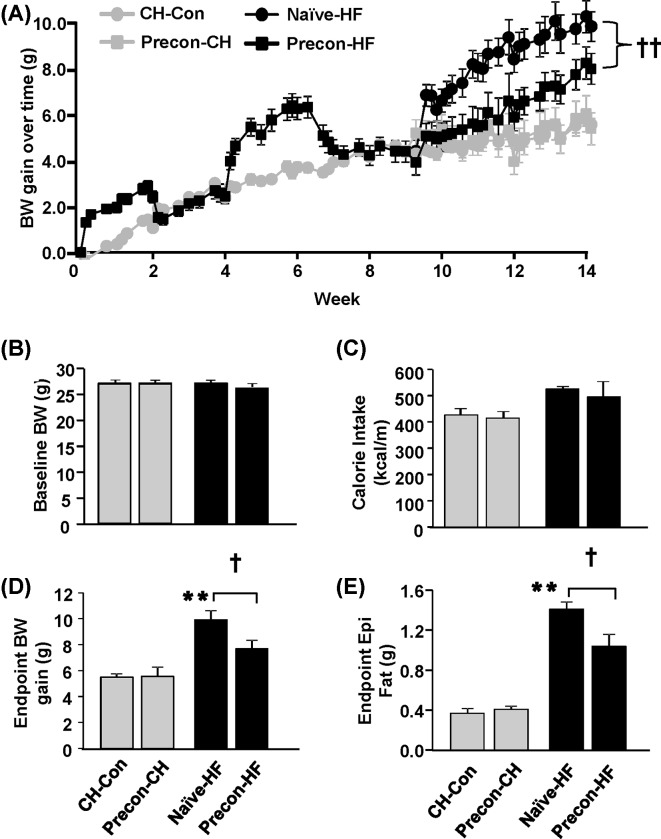
Effects of HF prior exposures on HF-induced BW gain and adiposity The effects were assessed during the final 6 weeks of HF feeding after 2 weeks of washout with CH diet following HF preconditioning. (**A**) BW gain over time from the baseline during the assessment period. (**B**) BW at the baseline before the final period of HF feeding. (**C**) Cumulative calorie intake during the first 8 days of metabolic assessment period. (**D**) BW gain from the baseline and (**E**) the weight of epididymal fat mass at the end of study. **P<0.01 compared with CH-Con; ^†^*P*<0.05, ^††^*P*<0.01 compared with indicated group (*n*=8–10/group).

During the first episode of HF feeding, mice fed with the HF diet gained twice as much BW as mice fed the CH diet (Figure 2A). When these mice were subsequently fed the CH diet as a washout, their BW reduced, and their BW gain was not different from the CH mice at the end of the 2 weeks of CH feeding. Similarly, as soon as the second episode of HF diet started, the BW of the mice began to increase, and their BW gain was twice that of their CH counterparts at the end of the second exposure. Within 2 weeks after they were switched back to the CH diet again, the body mass of the mice pre-exposed to HF (preconditioned) returned to the level of the CH mice (Figure 2B).

After the washout with CH, mice were fed either the HF diet again or kept on the CH diet to investigate the legacy effect in response to a subsequent exposure to the HF diet. After switching back to the CH diet, the BW gain of preconditioned mice kept on CH diet (Precon-CH) was similar to that of the control CH group (CH-Con) (Figure 2A). Interestingly, the preconditioned mice gained less BW during the subsequent feeding with the same HF diet (Precon-HF) than the mice exposed to the HF diet for the first time (Naïve-HF) despite similar food intake (Figure 2A,C). At the end of the experiment, the BW gain and the fat pad mass of the Precon-HF group was 22 ± 9% less and 26 ± 9% less respectively, compared with the Naïve-HF group (both *P*<0.05, Figure 2D,E). Together, these data indicate that the HF-preconditioning ameliorated the adiposity induced by subsequent exposure to the same HF diet, suggesting an induction of a legacy effect.

### Effects of HF prior exposures on HF-induced glucose and insulin intolerances

**Figure 3:**
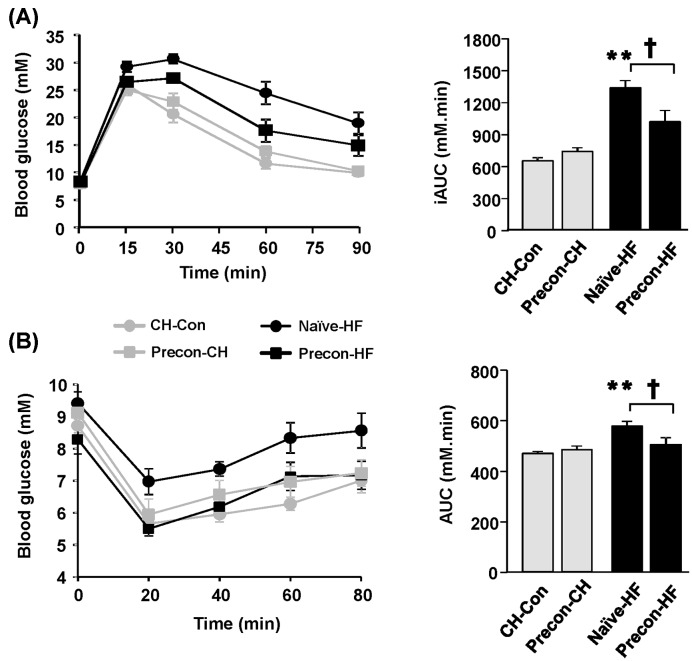
Effects of HF prior exposures on HF-induced glucose and insulin intolerances Glucose tolerance was performed by an ip injection of glucose at 2.0 glucose/kg BW (ipGTT) after week 2 of the final HF feeding (week 10). ITT was performed in following week (week 11) at a dose of 0.75 IU insulin/kg BW (ipITT). (**A**) Blood glucose levels during the ipGTT and its iAUC from 0–90 min (iAUC); (**B**) Blood glucose levels during the ipITT and its area under the curve from 0–90 min (AUC). **P<0.01 compared with CH-Con; ^†^*P*<0.05 compared with indicated group (*n*=7–9/group).

Insulin resistance is generally associated with obesity, and it is a major characteristic of type 2 diabetes. ipGTT and ipITT were performed to assess the legacy effect of preconditioning on whole-body glucose and insulin tolerance of mice. Compared with the CH-Con group, the Precon-CH had no changes in response to the glucose injection during ipGTT, as indicated by overlapping blood glucose curves (hence similar incremental area under the curve (iAUC)) (Figure 3A). However, when naïve or preconditioned mice were challenged with the HF diet, blood glucose levels of the Precon-HF mice were lower (27 ± 7%, *P*<0.05) at 60 min of the GTT and their iAUC values were less (23 ± 8%, *P*<0.05) than the Naïve-HF group. This indicates that preconditioning ameliorated HF-induced glucose intolerance.

To further assess the response of blood glucose levels to insulin stimulation, an ipITT was performed (Figure 3B). Again, the Precon-CH group showed a similar reduction in glucose levels and AUC in response to insulin injection compared with the CH-Con group. Although not statistically significant, blood glucose levels tended to be more reduced in the Precon-HF group compared with the Naïve-HF group at 20, 40, 60 and 80 min during the ipITT. Furthermore, the AUC of the Precon-HF group was 13 ± 5% less than the Naïve-HF (*P*<0.05). These results indicate that HF-preconditioning has protective effects against the HF-induced glucose intolerance and improves whole-body insulin sensitivity of HF mice.

### Effects of HFru prior exposures on HFru-induced adiposity

**Figure 4:**
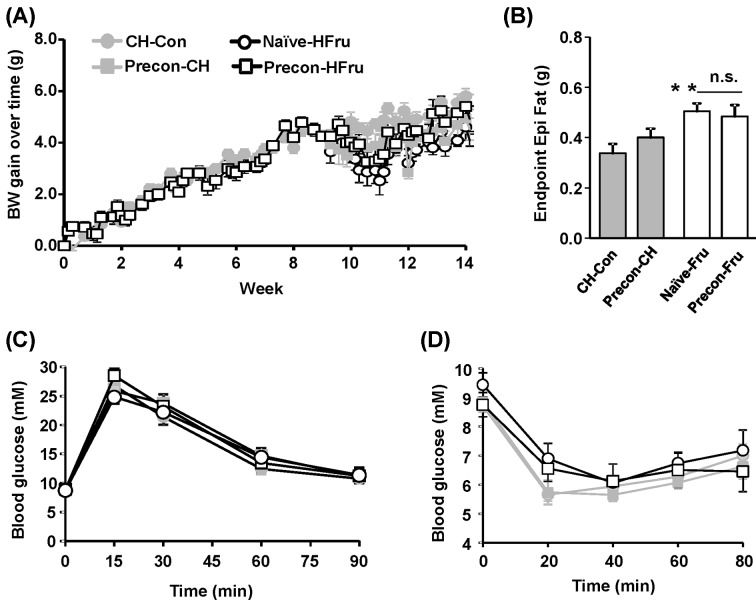
Effects of HFru prior exposures on HFru-induced adiposity The effects were assessed during the final 6 weeks of HFru feeding after 2 weeks of washout with CH diet following HFru preconditioning. (**A**) BW gain over time from the baseline during the assessment period. (**B**) The weight of epididymal fat mass at the end of study. Glucose tolerance was performed by an ip injection of glucose at 2.0 glucose/kg BW (ipGTT) after week 2 of the final HFru feeding (week 10). ITT was performed in following week (week 11) at a dose of 0.75 IU insulin/kg BW (ipITT). (**C**) Blood glucose levels during the ipGTT and its iAUC from 0–90 min (iAUC). (**D**) Blood glucose levels during the ipITT and its area under the curve from 0–90 min (AUC). ** *P*<0.01 compared with CH-Con. Abbreviation: n.s., not statistically significant (*n*=6–9/group).

Overconsumption of sucrose is known to cause the human metabolic syndrome largely from the derived fructose [22–25] and similar metabolic characteristics well resemble in HFru rodents including mice [16,26,27]. Thus, in a separate experiment, we fed mice with a HFru diet [16] under identical protocol for the HF-preconditioning experiment to examine whether a legacy effect may also occur in HFru-preconditioned group. Mice in four different groups (CH-Con, Precon-CH, Naïve-HFru, Precon-HFru) showed similar BW gain over the whole experiment period (Figure 4A). At the end of the experiment, there was no significance of epidydimal (Epi) fat weight between Naïve-HFru group and Precon-HFru group (0.51 ± 0.03 compared with 0.48 ± 0.05, *P*>0.5, Figure 4B). In addition, blood glucose levels were similar in four groups during ipGTT and ipITT (Figure 4C,D). These results indicate that these beneficial legacy effects are specific to HF diet, but not to HFru diet, suggesting a possible involvement of HF-derived factors. We then examined the effect of HF-preconditioning on liver, muscle and adipose tissues, the key organs for energy metabolism and glucose homoeostasis.

### Effects of HF prior exposures in liver

**Figure 5:**
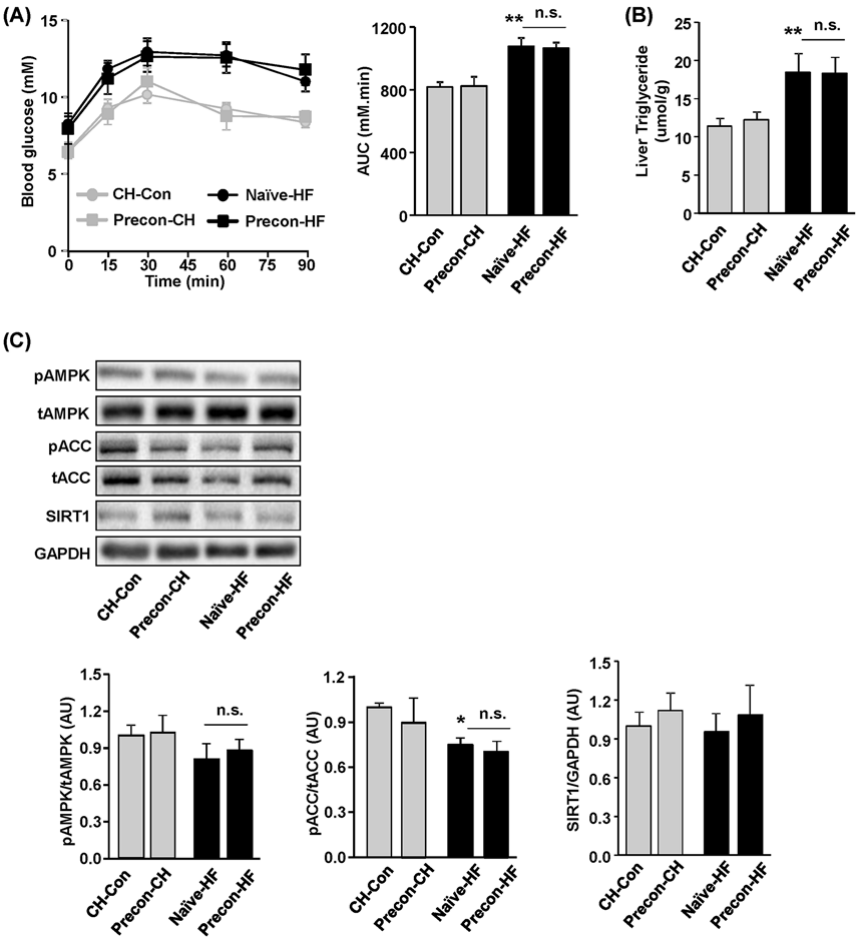
Effects of HF prior exposures in liver The effects of preconditioning in liver were assessed by PTT and examination of liver tissue. (**A**) Blood glucose levels during the ipPTT (2.0 g pyruvate/kg BW by an ip injection) assessed after 4 weeks of the final HF feeding (week 12) and blood glucose AUC (*n*=6–9/group). (**B**,**C**) Liver samples were freeze-clamped at the end of the study (week 14) for the following measurements. (B) TG content; (C) protein levels of pAMPK, tAMPK, pACC, tACC, and SIRT1 were determined by Western blotting and normalised by GAPDH (*n*=6–7/group). *P<0.05, **P<0.01 compared with CH-Con. Abbreviation: n.s., not statistically significant.

As one of the major tissues in the regulation of glucose, the liver plays an important role in maintaining glucose homoeostasis via gluconeogenesis from substrate metabolites such as pyruvate and lactate. The PTT measures hepatic glucose production from the injected pyruvate through gluconeogenesis. It can be used to determine whether there is an increase in the capacity of hepatic glucose production. As shown in Figure 5A, the glucose levels and AUC during the PTT in preconditioned mice were similar to those in naïve mice, either between the Precon-CH and CH-Con group or between Precon-HF and Naïve-HF group. This suggests that HF-preconditioning did not significantly affect the capacity for glucose production in the liver.

Next, the effect of HF-preconditioning on hepatic steatosis was assessed by measuring TG content in the liver. As shown in Figure 5B, when mice were fed either CH or HF, preconditioning did not change the TG content in the liver compared with mice that had not been preconditioned. This suggests that protection against obesity through HF-preconditioning was not related to changes in hepatic steatosis.

AMPK plays a pivotal role in the suppression of glucose production and promotion of fatty acid (FA) oxidation and mitochondrial biogenesis in the liver [28,29]. SIRT1 is reported to promote FA oxidation through the activation of PPARα in the liver [30] and regulate the activity of AMPK [31]. However, consistent with the results of ipPTT and TG content in the liver, no apparent change was observed on the activation of AMPK and its downstream target, ACC (Figure 5C). Again, no apparent change of the protein level of SIRT1 was observed by HF-preconditioning (Figure 5C).

### Effects of HF prior exposures in muscle

**Figure 6:**
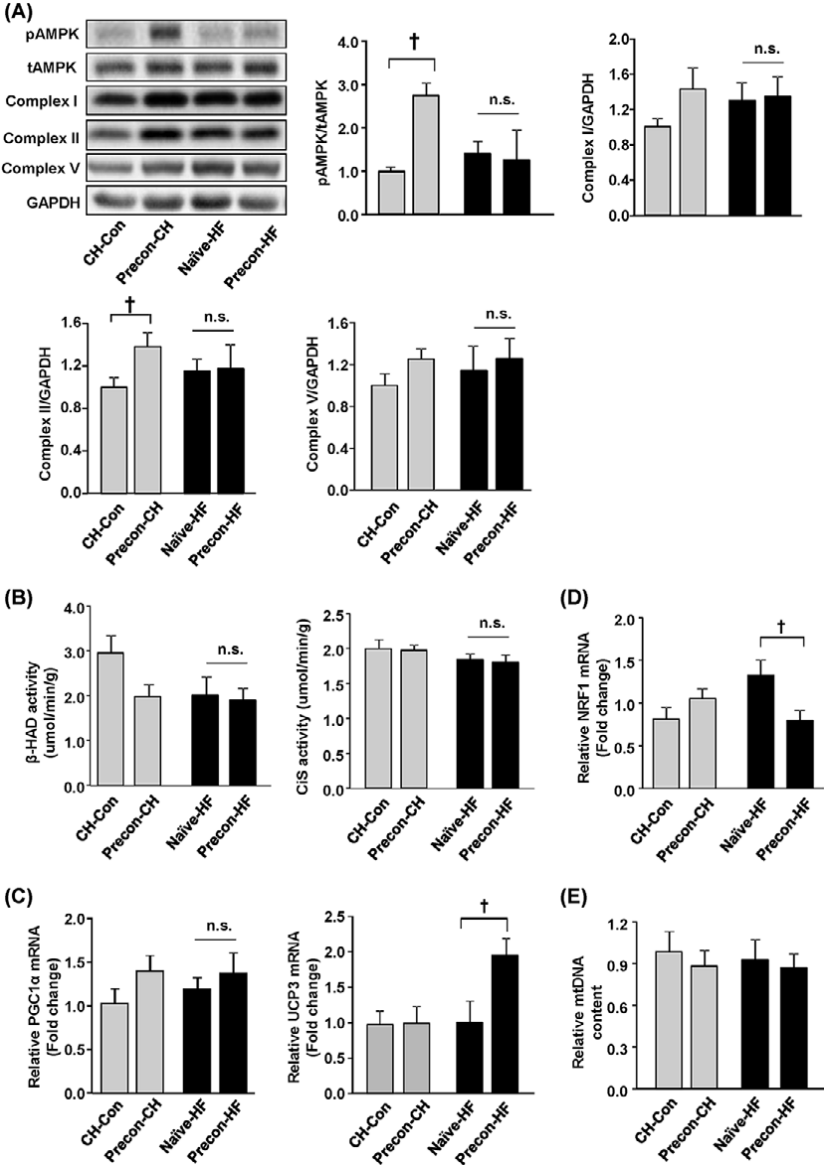
Effects of HF prior exposures in muscle Quadriceps muscle was collected from the mice at the end of the study (week 14) for the following measurements. (**A**) Protein levels of AMPK and mitochondrial complex measured by Western blotting and normalised by GAPDH; (**B**) β-HAD activity was measured by the disappearance rate of NADH, and CiS activity (CiS) was detected by detecting the rate of the CoA-SH production using DTNB. (**C**) Levels of mRNA expression of PGC1α and UCP3 and (**D**) nuclear respiratory factor-1 (NRF1) were quantified by qRT-PCR. (**E**) mtDNA content was measured by quantificative PCR using MCT-1 (genomic DNA marker) as a control gene. ^†^*P*<0.05 compared with indicated group (*n*=8–10/group).

Skeletal muscle is a major contributor to the resting energy expenditure mainly through mitochondrial oxidation of energy-rich substrates such as FAs. FA oxidation has also been linked to the development of insulin resistance in this tissue [32,33]. The capacity of mitochondrial oxidative phosphorylation was examined to evaluate the effects of preconditioning on muscle using the samples collected at the end of the study. Although mitochondrial complex II in the Precon-CH group increased by 38 ± 13% compared with the CH group (*P*<0.05), complexes I and V were not affected by preconditioning (Figure 6A). Meanwhile, β-HAD and CiS are two mitochondrial enzymes involved in mitochondrial FA oxidation and their activities are often used as indicators of mitochondrial function [16,34]. The results showed that their activities were not changed by preconditioning (Figure 6B).

Further analyses were conducted with muscle samples by measuring relevant transcription factors involved in the regulation of mitochondrial biogenesis, including mRNA expressions of PGC1α and UCP3 together with pAMPK/total AMPK protein levels. There was a two-fold increase in AMPK phosphorylation in Precon-CH mice (*P*<0.05 compared with CH), but no apparent change in Precon-HF mice (*P*>0.1 compared with Naïve-HF) (Figure 6A). No significant change in *PGC1α* mRNA expression was observed between naïve and preconditioning groups (Figure 6C). However, the mRNA expression of this protein was decreased by 50 ± 12% in Precon-HF group than the Naïve-HF group (*P*<0.05). The Precon-HF group showed a significant increase in mRNA expression level of UCP3 than the naïve-HF group (*P*<0.05, Figure 6C). A similar trend was observed in the mRNA expression of nuclear respiratory factor-1 (NRF1) (Figure 6D). As no consistent effect of preconditioning on the regulation of mitochondrial biogenesis was observed, total mtDNA content was measured to investigate whether the number of mitochondria was affected by preconditioning. The results showed that preconditioning did not affect the mtDNA content in muscle (Figure 6E).

### Effects of HF prior exposures on plasma FGF21 and metabolic capacity in adipose tissue

**Figure 7:**
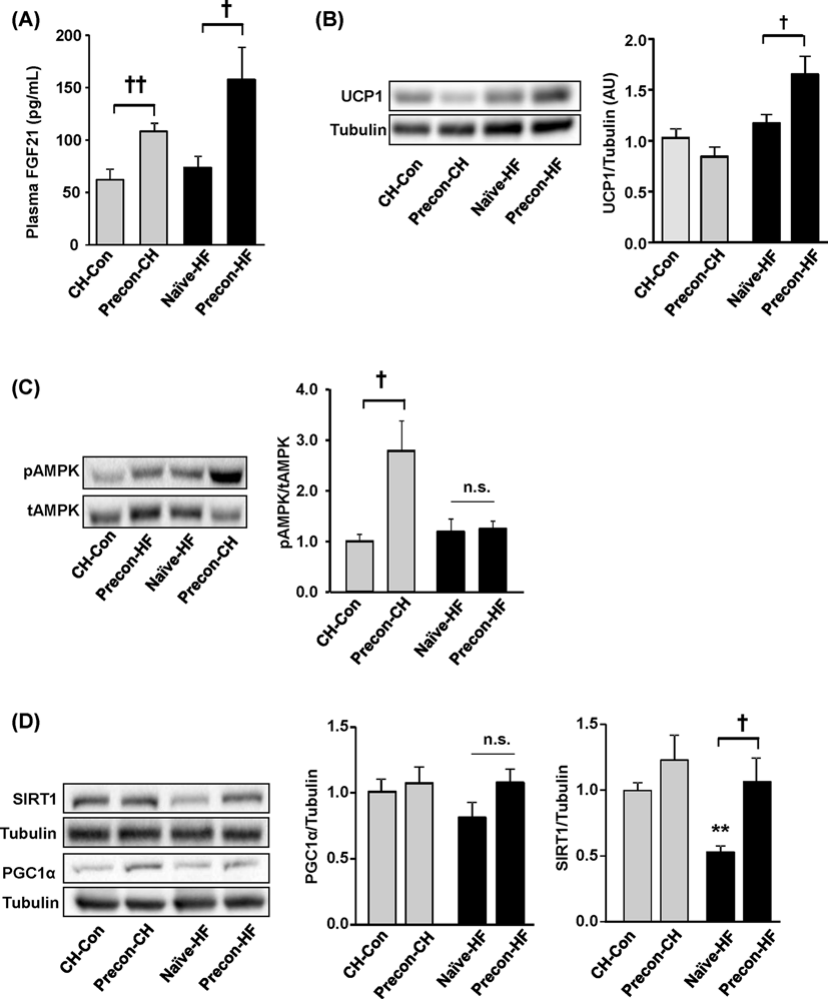
Effects of HF prior exposures on plasma FGF21 and metabolic capacity in adipose tissue Epididymal fat was collected from the mice at the end of the study (week 14) for the following measurements. (**A**) Plasma levels of FGF21 were measured by ELISA at the end of the study. (**B**–**D**) UCP1, AMPK, PGC1α and SIRT1 in epididymal adipose tissue measured by Western blotting at the end of the study. **P<0.01 compared with CH-Con;^†^*P*<0.05, ^††^*P*<0.01 compared with indicated group (*n*=6–10/group).

FGF21, an emerging regulator of energy expenditure, has been shown to increase the energy consumption through multiple mechanisms including the ‘browning’ in white adipose tissue (WAT), which is partly through increasing PGC1α protein content [35]. An elevation in circulating FGF21 was observed in Precon-CH group compared with CH-Con group (*P*<0.01) and a significant increase in Precon-HF group compared with Naïve-HF (*P*<0.05) (Figure 7A), suggesting the preconditioning of HF diet increased FGF21 level in the blood. In addition, increased expression of UCP1 is a hallmark of ‘browning’. While no significant increase in the protein level of UCP1 was observed in the Precon-CH group, there was a 54 ± 20% increase in the Precon-HF group compared with the Naïve-HFC group (*P*<0.05, Figure 7B). Interestingly, we observed an increase in pAMPK in Precon-CH group compared with CH-Con. However, when we challenge mice with HF diet again (Precon-HF), this increase disappeared (Figure 7C). PGC1α is a key transcription factor that regulates gene expression involved in the ‘browning’ [36]. However, there was no apparent change in the mRNA expression levels of PGC1α with preconditioning regardless the subsequent CH or HF challenge (Figure 7D). It has been suggested that ‘browning’ of WAT can be regulated by SIRT1 [37]. A significant increase in SIRT1 protein levels was observed in Precon-HF compared with the Naïve-HF group and a similar trend was observed in Precon-CH group compared with the CH-Con group (Figure 7D).

## Discussion

The present study investigated whether prior exposures to HF diet in the adulthood may induce long-lasting risk to the development of the metabolic syndrome. Based on the reported risk of the early life experience like this [10–13], we had hypothesised that prior feeding with an HF diet would exacerbate the metabolic syndrome in the subsequent response to the HF diet. In opposite to our initial hypothesis, the results showed that exposures to HF diet in adult mice actually blunted the severity of BW gain, central adiposity and glucose intolerance. This finding indicates that prior HF exposures induce mechanisms of adaptation to resist the metabolic syndrome, conceptually similar to the preconditioning against ischaemic injury in heart [38]. Because the metabolic syndrome during the prior HF exposures had been washed out, the sustained protective effects can be considered as the legacy effects as described after certain interventions [5,6]. It is also interesting to note that the observed protection effects only occurred in prior HF exposures (preconditioning) but not occurred in HFru diet, which induces the metabolic syndrome from hepatic steatosis by intrinsic lipogenesis [39].

HF feeding in mice can induce obesity and associated metabolic disorders which have been well characterised to resemble the metabolic syndrome in humans from overconsumption of fat [40–42]. As HF-fed mice can develop obesity, glucose intolerance and hepatic steatosis within 2 weeks [40,43], the present study set 2 weeks to induce the full metabolic effects for the two episodes of HF prior exposures (or preconditioning). As expected, the effectiveness of the HF preconditioning was demonstrated clearly in the present study by an increased BW gain during each period of the preconditioning. To examine the legacy effects separately from the metabolic syndrome directly produced by HF preconditioning, the metabolic responses were assessed after 2-week washout by a normal low-fat diet, namely CH. Indeed, the direct metabolic effects (as indicated by BW gain) of HF diet during the preconditioning period were reversed to the normal after switching to the CH diet for 2 weeks. Following these prior treatments, the metabolic responses to the HF diet during the subsequent period of 6 weeks represent the legacy effects in a reasonably long-term in adult mice.

Unlike the reported detrimental legacy effects of HF diet in parents on the offspring [44] or from prenatal to adult [45], the present study revealed the protective effect of HF preconditioning (Precon-HF group) in reducing the BW gain response to subsequent HF feeding compared the mice without HF preconditioning (Naïve-HF group). Consistent with this, visceral fat mass, iAUC during ipGTT and AUC during ipITT in Precon-HF mice were significantly lower than the Naïve-HF group. These results suggest that the mechanisms involved in the legacy effects of HF preconditioning in the same generation of the adult are different from those underlying legacy effects on the offspring exposure to HF diet to its parent generation. For example, the legacy effects displayed in the offspring as a result of HF prior exposure in the parent are believed to result from epigenetic alterations in the reproductive cells [44]. Similarly, the impact of perinatal factors to the adulthood is likely to involve the change in organ/tissue development via altered genetic programming [46]. Because the present studies were conducted in mature adult mice, it is likely that the mechanisms lie in the organs directly responsible for the homoeostasis of energy balance and glucose metabolism.

Since the observed protective effects occurred without a significant change in caloric intake, mechanisms underlying the protective effects of preconditioning on obesity and glucose metabolism, relevant parameters indicative of changes in metabolism in the liver, muscle and adipose tissue (major peripheral tissues responsible for energy metabolism and glucose homoeostasis) were examined.

In the liver, there was no improvement in either capacity of glucose production (indicated by an unchanged response to a PTT) or hepatic steatosis (indicated by unchanged TG content) between Precon-HF group and Naïve-HF group. In addition, the activities of AMPK and SIRT1 did not show apparent changes. These results might suggest that the liver does not play a significant role in mediating the beneficial legacy effects of HF diet. This interpretation appears to be consistent with the lack of protective effects of preconditioning with HFru diet in the present study because liver is the primary organ in HFru-induced metabolic syndrome [39].

Next, the markers involved in mitochondrial oxidative phosphorylation and FA metabolism were measured as an indication of the capacity of energy metabolism in the muscle. The overall mitochondrial content (indicated by mtDNA content) and activities of enzymes involved in mitochondrial FA oxidation were not affected by preconditioning, despite increases in AMPK activation and mitochondrial complex II in Precon-CH mice. The reason for this discrepancy is unknown and further studies are needed to determine whether skeletal muscle contributes to the protective effects of preconditioning.

UCP1 mediates thermogenesis in adipose tissue and increases energy expenditure. It is expressed in brown adipose tissue or brown-like adipocyte within WAT as a marker of browning [1,47,48]. Interestingly, UCP1 protein levels were increased in WAT of the Precon-HF group. Consistent with the increased UCP1 protein levels, Precon-HF group had increased levels of circulating FGF21, a key inducer of the ‘browning’ [35], and increased levels of SIRT1 protein in the WAT. It has been reported that FGF21 and SIRT1 increase the level of PGC1α in WAT respectively, through post-transcriptional mechanisms [35,37]. Although no apparent change in mRNA expression of PGC1α was observed, further measurement of the protein levels of these two transcription regulators are needed to indicate their possible involvement in the preconditioning-induced ‘browning’. In addition, it has been suggested that subcutaneous WAT is more sensitive to FGF21 than the epididymal WAT used in this study [35,49]. This is associated with an increase in UCP1 and SIRT1 levels in WAT as well as an increase in circulating levels of FGF21, which, we speculate, may be involved in the ‘browning’ of WAT as a possible mechanism. Because the circulating FGF21 is mainly released from the liver through the activation of PPARα [50,51], we also speculate that the ‘browning’ in WAT may be indirectly induced by PPARα in the liver. Obviously further research is needed to investigate this possibility by blocking PPARα such as using PPARα knockout mice or eliminating FGF21 such as using FGF21 knockout mice.

It is also worth pointing out that the present study preconditioned mice with two episodes of HF in an attempt to boost the metabolic memory of the legacy effects based on the protocols used some immunisations. Future studies need to investigate whether the observed protective legacy effects may be induced by one single episode of HF preconditioning and whether the immune system may also be involved. The findings from the present study also provide a basis for further investigation of various manipulations of dietary regiments such as the legacy effects of HF preconditioning on HFru-induced metabolic syndrome or *vice versa*. Such studies would shed more lights on the mechanism underlying the protective legacy effects observed in the present study.

In summary, the present study shows that HF-preconditioning in adult mice ameliorates the development of obesity and glucose intolerance in response to subsequent feeding of HF diet. The mechanisms of these protective legacy effects involve the enhancement of metabolic capacity in skeletal muscle and WAT. Similar to the concept of preconditioning protection against ischaemic injury in heart [38], the paradoxical metabolic phenomenon revealed in the present study may provide novel insight into the identification potential new drug targets for the treatment of obesity and related metabolic disorders in humans. Additionally, the breakdown products of an HF diet during the intermittent prior exposures may produce by short-chain FAs or ketone bodies which are have been suggested to promote energy metabolism and induce FGF21 [52]. We speculate that these breakdown products from HF-preconditioning can also be explored for as possible dietary manipulations to help control the metabolic syndrome in humans.

## Abbreviations

ACC: acetyl-CoA carboxylase
AMPK: AMP-activated protein kinase
BW: body weight
CH: chow
CiS: citrate synthase
DTNB: 5,5′-dithiobis(2-nitrobenzoic acid)
FA: fatty acid
FGF21: fibroblast growth factor 21
GTT: glucose tolerance test
HF: high fat
HFru: high fructose
HRP: horseradish peroxidase
iAUC: incremental area under the curve
ip: intraperitoneal
ipPTT: ip pyruvate tolerance test
ITT: insulin tolerance test
MCT-1: monocarboxylate transpoter 1
ND-5: NADH-ubiquinone oxidoreductase chain 5
PCG1α: peroxisome proliferator-activated receptor gamma coactivator 1 alpha
PPARα: peroxisome proliferator-activated receptor alpha
PTT: pyruvate tolerance test
SIRT1: Sirtuin 1
TG: triglyceride
UCP1: uncoupling protein 1
UCP3: uncoupling protein 3
WAT: white adipose tissue
β-HAD: β-hydroxyacyl-CoA dehydrogenase
